# Coevolution Trumps Pleiotropy: Carbon Assimilation Traits Are Independent of Metabolic Network Structure in Budding Yeast

**DOI:** 10.1371/journal.pone.0054403

**Published:** 2013-01-10

**Authors:** Dana A. Opulente, Christopher M. Morales, Lucas B. Carey, Joshua S. Rest

**Affiliations:** 1 Department of Ecology and Evolution, Stony Brook University, Stony Brook, New York, United States of America; 2 Department of Computer Science and Applied Mathematics, Weizmann Institute of Science, Rehovot, Israel; The Scripps Research Institute, United States of America

## Abstract

Phenotypic traits may be gained and lost together because of pleiotropy, the involvement of common genes and networks, or because of simultaneous selection for multiple traits across environments (multiple-trait coevolution). However, the extent to which network pleiotropy versus environmental coevolution shapes shared responses has not been addressed. To test these alternatives, we took advantage of the fact that the genus *Saccharomyces* has variation in habitat usage and diversity in the carbon sources that a given strain can metabolize. We examined patterns of gain and loss in carbon utilization traits across 488 strains of *Saccharomyces* to investigate whether the structure of metabolic pathways or selection pressure from common environments may have caused carbon utilization traits to be gained and lost together. While most carbon sources were gained and lost independently of each other, we found four clusters that exhibit non-random patterns of gain and loss across strains. Contrary to the network pleiotropy hypothesis, we did not find that these patterns are explained by the structure of metabolic pathways or shared enzymes. Consistent with the hypothesis that common environments shape suites of phenotypes, we found that the environment a strain was isolated from partially predicts the carbon sources it can assimilate.

## Introduction

A goal of evolutionary biology is to understand the selective pressures that shape variation in genomes and phenotypes [1]–[7]. Little is known about the evolutionary forces that shape the suite of carbon sources that an organism can utilize in metabolism. We propose two hypotheses that shape common utilization and loss: (1) carbon assimilation traits are gained and lost together because sets of carbon sources are common to particular environments, or (2) sets of carbon assimilation traits are gained and lost together because the processing of carbon sources often share common metabolic pathways. The first hypothesis means that multiple traits have coevolved because of patterns of similarity across environments [8], while the latter hypothesis implies that pathway or gene-based pleiotropy drives the coordinated gain and loss of multiple traits [9], [10]. The diversity of carbon sources used by *Saccharomyces* provides a unique opportunity to study the patterns of gain and loss in carbon utilization and evaluate how these patterns are related to the structure of the metabolic networks and to each strain's environmental source.

Strains in the genus *Saccharomyces* are found in a range of habitats including soil, plants, fruits, fish, and insects. Correspondingly, *Saccharomyces* strains can utilize a diverse range of carbon sources. Carbon sources metabolized within the genus include simple sugars, polyols, organic and fatty acids, aliphatic alcohols, hydrocarbons, and various heterocyclic and polymeric compounds [11]. However, not all strains can use all of these carbon sources.

We compiled growth data from the CBS-KNAW Fungal Biodiversity Centre for strains in the genus *Saccharomyces* to systematically assess patterns of covariation, gain, and loss in carbon utilization. We find that subsets of carbon traits that are gained and lost together cannot be explained by shared metabolic pathways or shared enzyme use. In contrast, we did find that the environment a strain was isolated from partially predicts the set of carbon sources it may assimilate and metabolize. Together, these results suggest that selection by environmental factors may often trump pleiotropy in shaping covariation in sets of carbon assimilation traits.

## Methods

### Cataloging carbon utilization phenotypes

Growth phenotypes across multiple carbon sources and strain origin data for 448 strains in the genus *Saccharomyces* were retrieved from CBS-KNAW Fungal Biodiversity Centre [12]. We only considered carbon sources that were tested in at least 200 strains and only strains that were tested for at least 20 carbon sources. Either a normal or weak growth phenotype, as reported in CBS-KNAW, was considered evidence for utilization of a particular carbon source.

Of possible growth phenotypes across strains, growth data for 8% of the strains were missing in the dataset. For this missing data, we performed a simple random data imputation to infer the carbon utilization trait.

We tested whether growth phenotypes for a carbon source showed an overrepresentation for weak or strong growth using a χ^2^-test. If there was no bias for a specific growth phenotype, we would expect similar numbers of strains displaying either a weak or strong growth phenotype. However, if there was a bias for growth phenotype, the observed data would deviate from similar numbers of weak and strong growth phenotypes.

### Carbon source utilization cluster analyses by pathway and enzyme

We assessed whether gains and losses of carbon sources cluster by strain using multiscale bootstrap resampling, with 1000 permutations (R v2.14 package pvclust v1.2–2). We produced a matrix between carbon sources reflecting ability to be utilized by the same strains. Each carbon source was then assigned a cluster and similar clusters were joined together until there was only a single cluster remaining. To assess if these patterns were driven by overlapping metabolic pathways, pathway data for each carbon source was acquired from the Kyoto Encyclopedia of Genes and Genomes (KEGG) v62.0 [13]. Carbon sources were clustered by Ward's method (also with pvclust in R) according to their presence or absence in each metabolic pathway or based on direct interactions with enzymes.

### Isolation predicted by carbon sources

We assessed whether carbon source use patterns were driven by a common environment by predicting strain isolations based on carbon source sets. We used a k-nearest neighbor classification (k = 3) with a leave-one-out cross validation scheme to determine if carbon source sets could be used to predict strain isolation (MATLAB 2012b).

## Results

### Carbon source utilization diversity

**Figure 1:**
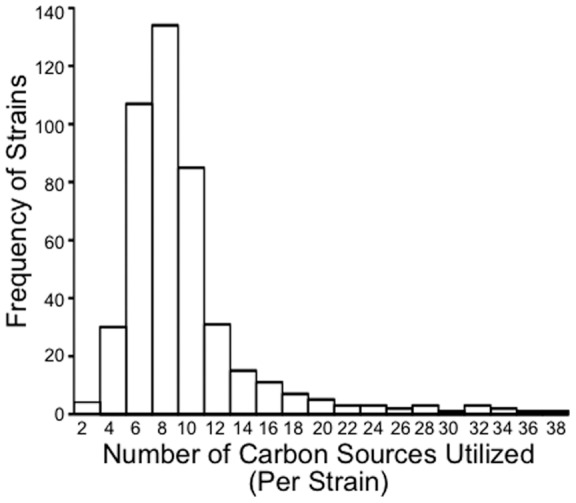
The genus *Saccharomyces* displays variation in the number of carbon sources that each strain can utilize. Growth data was obtained from CBS-KNAW [12].

Carbon utilization is diverse within the genus *Saccharomyces*. On average, strains can grow on approximately 8 carbon sources (*Table 1*); however strains can use a range of 1 to 37 carbon sources (Figure 1).

**Table 1:** Summary statistics for the number of carbon sources with normal and weak growth phenotypes across 448 strains of *Saccharomyces*.

Statistic	Normal Growth	Weak Growth	Total Growth	No Growth
Mean	7.04	1.88	8.92	37.83
Median	7	0	8	37
S.D.	2.97	4.31	5.20	5.76

**Figure 2:**
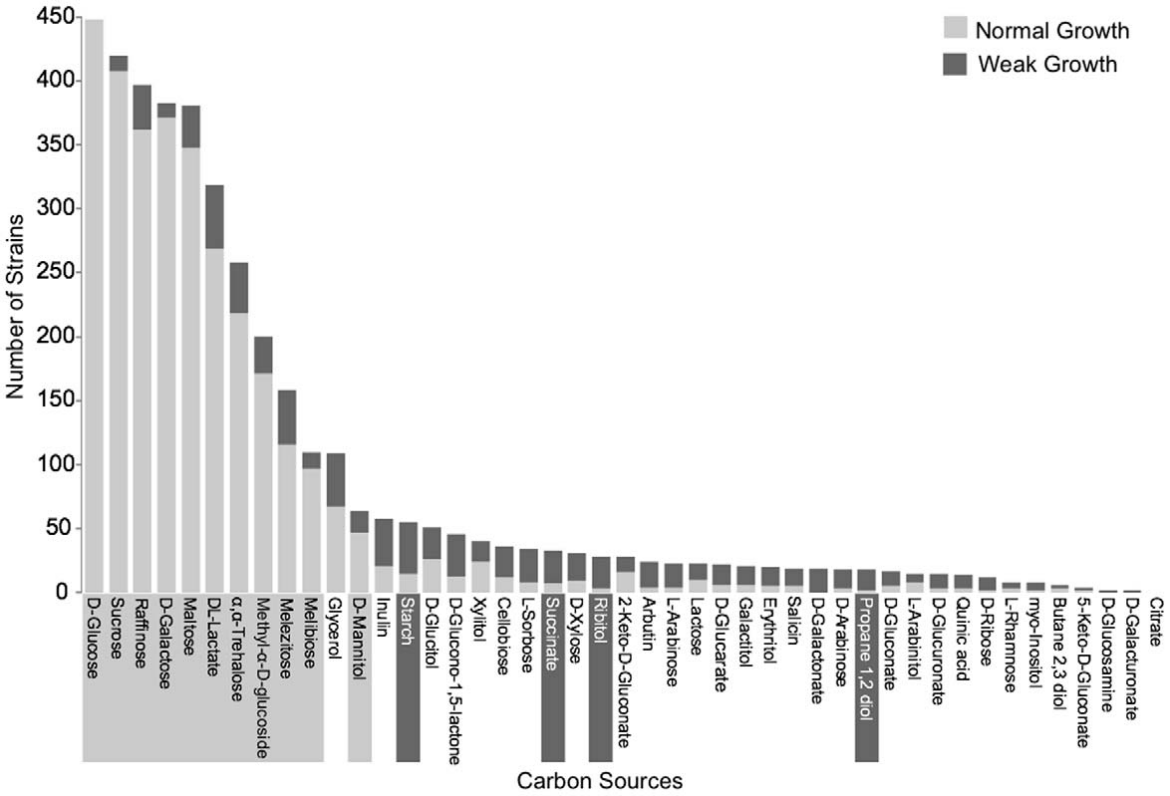
Strains show a bias for weak or normal growth phenotypes for 15 carbon sources. The light and dark grey bars represent weak and normal growth phenotypes, respectively. The light and dark grey boxes surrounding the carbon source names represent carbon sources which display an overrepresentation for a normal or weak growth phenotype, respectively, across all strains (χ^2^-test; p<0.001).

*Saccharomyces* strains differ in their growth rate on most carbon sources. In the data analyzed here, strains display either a normal or weak growth phenotype on each carbon source. On average, strains grow normally on 7 carbon sources and grow weakly on an additional 1.88 carbon sources (*Table *1). All strains grow normally on glucose. To test whether some carbon sources are more likely to result in a slow versus normal growth phenotype, we examined the association between carbon sources and growth rate phenotype. Out of the 45 tested carbon sources, 11 carbon sources are overrepresented for normal growth across strains, relative to a weak growth phenotype (χ^2^-test, p<0.001, indicated with a light gray boxes in Figure 2). For example, all 488 strains display a normal growth phenotype on glucose, indicating that glucose is overrepresented for the normal growth phenotype (p = 1.96×10^−99^). This over-representation is expected, as glucose is the preferentially used carbon source of *S. cerevisiae* and other species in the genus [14]. Additional carbon sources which display an overrepresentation for the normal growth phenotype include sucrose (p = 3.45×10^−83^), D-galactose (p = 3.68×10^−75^), α,α-trehalose (p = 1.53×10^−28^), and maltose (p = 1.38×10^−58^).

In contrast, 4 carbon sources show an overrepresentation for a weak growth phenotype, relative to normal growth phenotype: starch (p = 7.48×10^−4^), succinate (p = 9.41×10^−4^), ribitol (3.21×10^−5^), and propane 1,2 diol (p = 9.67×10^−4^) (dark gray boxes in Figure 2). For example, of the 55 strains that can use starch, 40 display a weak growth phenotype. This over-representation of weak growth is consistent with previous work showing that while starch can be used by *S. cerevisiae* and other species, they are inefficient at hydrolyzing starch [15], [16].

### Patterns of gain and loss in carbon utilization

The diversity found for carbon use traits raises the question – is there covariance for gain and loss in carbon utilization? In other words, are strains that have gained or lost the ability to use a particular carbon source more or less likely to have gained or lost the ability to use other carbon sources? There are two mechanisms by which the ability to use particular carbon sources may be gained and lost. First, different environments contain consistently different sets of carbon sources, and strains adapted to alternative environments may be enriched or depleted for respective carbon utilization traits (common environment hypothesis). Second, the metabolism of related carbon sources is achieved by multifunctional enzymes or through alternative steps or configurations of the same metabolic pathways. In these cases, selection for gain or loss of one carbon source may cause concomitant gain or loss of related carbon sources (common network hypothesis).

**Figure 3:**
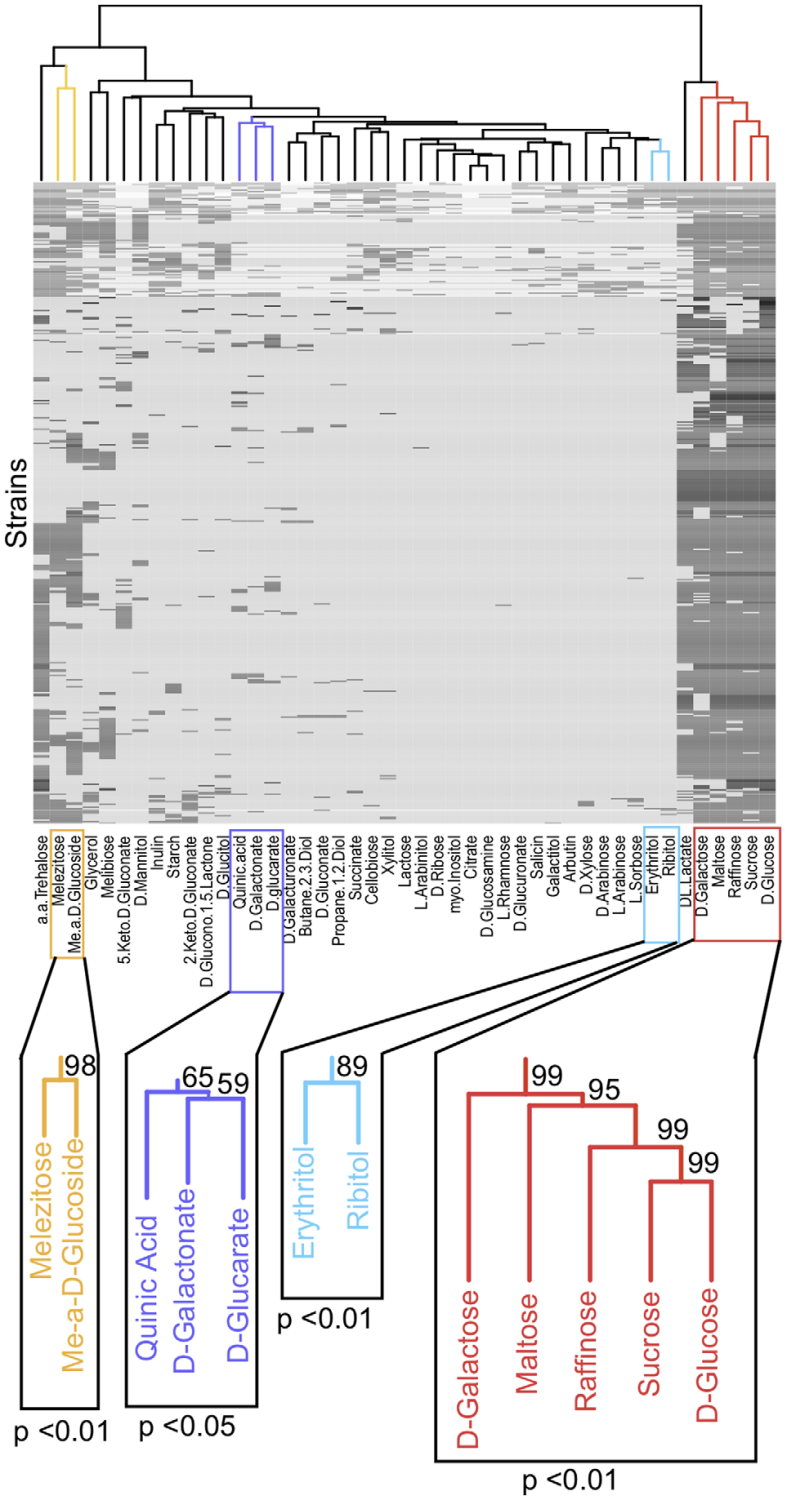
Nonrandom distribution of four clusters of carbon source utilization traits across strains. The heat map shows the ability (black cells) or inability (grey cells) of each strain to utilize each carbon source. Missing data is indicated by white cells. Callout boxes indicate significant clusters of carbon sources according to multiscale bootstrap resampling. P-values and bootstrap support are provided for the significant clusters.

If carbon source use has been gained and lost in groups, we predict that the distribution of carbon utilization traits will be non-random across diverse *Saccharomyces* strains and species. To test this prediction, we used a multiscale bootstrap analysis to assess whether these carbon utilization traits are distributed non-randomly among strains. Most carbon sources were gained and lost independently of each other. However, we found 4 clusters, involving 2 to 5 carbon sources each, for which gains and losses of carbon sources are significantly associated with each other (Figure 3).

We tested whether common networks are associated with these non-random gains and losses of carbon utilization traits by examining the distribution of carbon gain and loss on the yeast metabolic network. If multiple carbon sources are used in the same pathway, those traits can be gained or lost together through the addition or removal of any node in that pathway. Alternatively, carbon utilization traits may be related only by overlap of just a single enzyme in the pathway [17]. In either of these cases, carbon sources that require the same enzymes will cluster together in carbon utilization patterns. Metabolic network data was collected from KEGG for all carbon sources analyzed in the strain data (i.e. in Figure 3), and clustering of carbon sources by metabolic pathway or shared enzyme was analyzed with hierarchical clustering.

**Figure 4:**
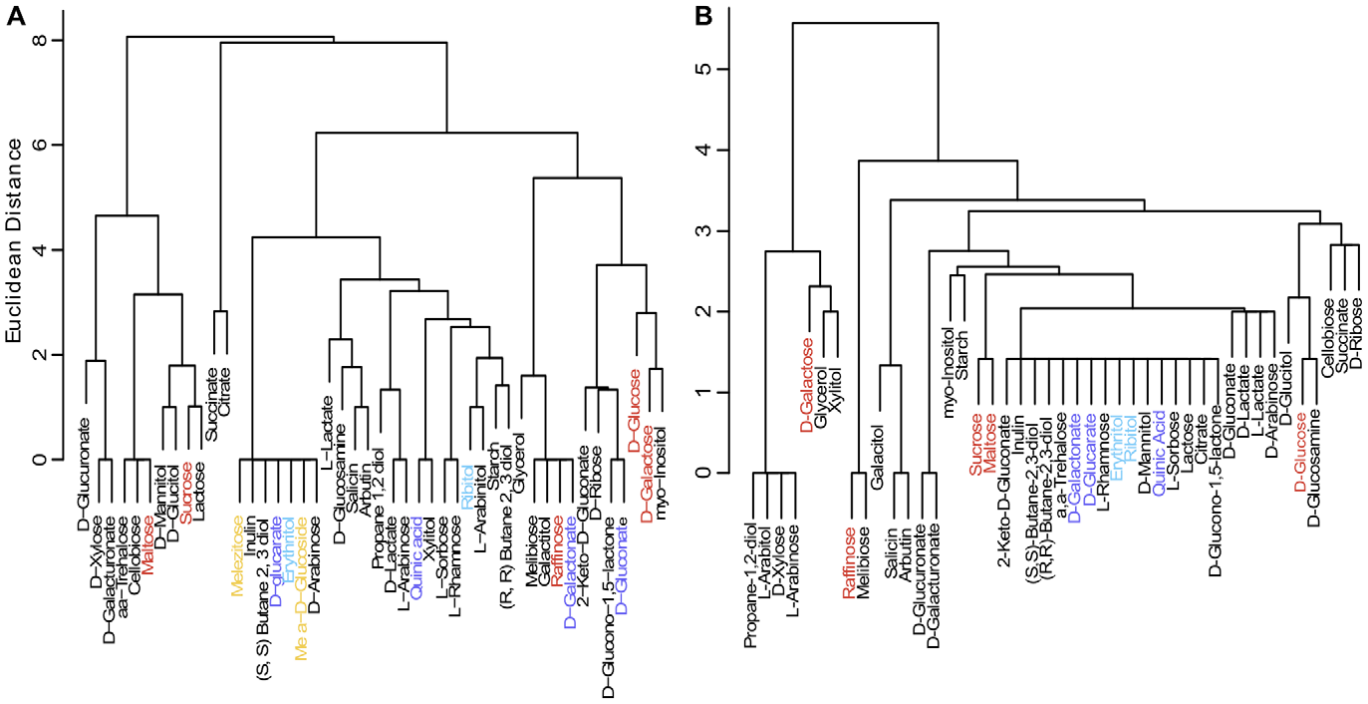
The clustering of carbon sources by (A) their metabolic pathways and (B) enzymes they directly interact with are each different from the clustering of carbon sources by their utilization across strains. The coloring of leaves in **A** and **B** corresponds to carbon sources from significant clusters in Figure 3. The lack of correspondence between these analyses suggests that patterns of gain and loss of carbon utilization traits are not driven by the metabolic pathways in which they are found. Clustering was performed by Ward's method.

**Figure 5:**
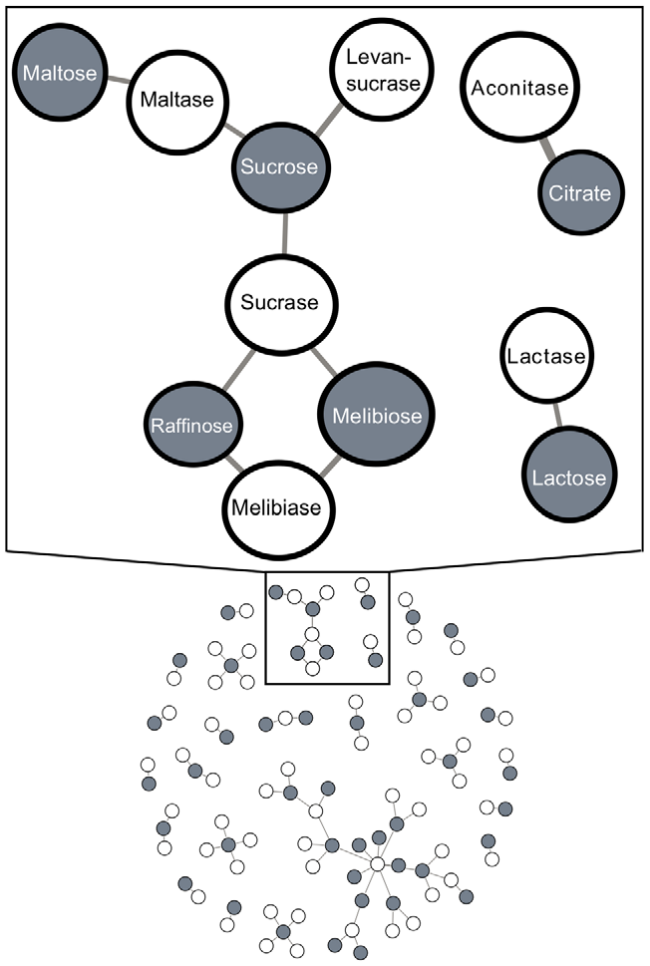
Most enzymes are specialized for catabolism of one or a few carbon sources. White nodes indicate enzymes and gray nodes indicate carbon sources. Edges represent the enzymes that process each carbon source.

In contrast to the common network hypothesis, we find no evidence that the structure of the metabolic network drives patterns of carbon utilization traits. Comparing the sister carbon sources in Figure 3 (carbon source clustered by strain) to the pattern of carbon sources clustered by metabolic pathways (Figure 4A), we find different carbon sources cluster together in the two analyses. The same is true for enzyme overlap: coordination of carbon utilization with enzyme overlap was also not observed (Figure 4B). Some carbon sources are associated with the same enzymes (Figure 5), but the majority of enzymes are specialized for single carbon sources. The dramatic covariation we see for carbon utilization is thus likely associated with gain and loss of specialized enzymes across strains. Such gain and loss may result in different network properties among strains (e.g. degree distribution).

**Figure 6:**
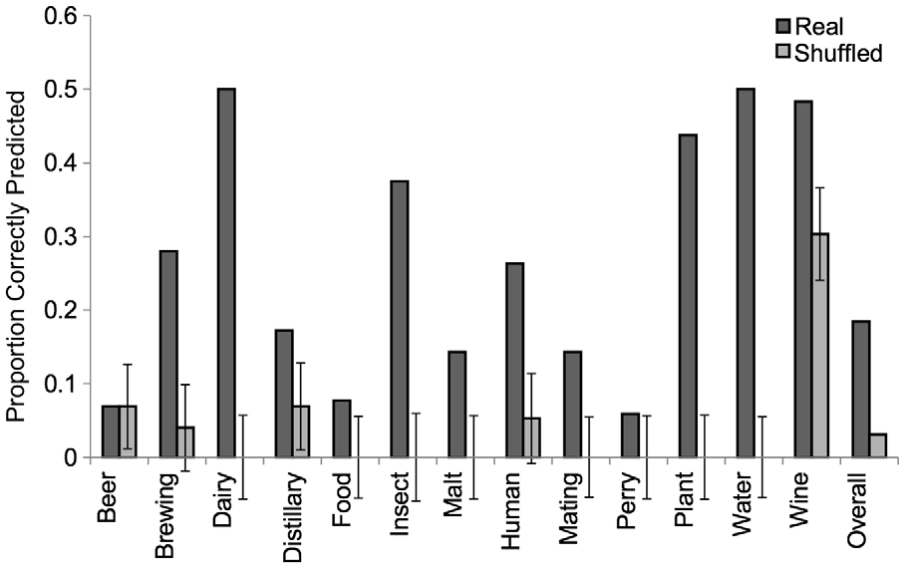
The substrate a strain was isolated from can be predicted based on the set of carbon sources a strain can utilize. Dark gray bars represent the proportion of time that a substrate was correctly predicted. Lighter gray bars represent the background prediction rate and associated confidence intervals based on shuffled data. There was no predictive power for 6 substrates (not shown).

In order to test whether patterns of carbon source gain and loss are associated with common environments, we analyzed whether the carbon source utilization profile of each strain can predict the natural substrate from which each strain was isolated. If strains isolated from similar environments have gained and lost the ability to grow on similar carbon sources, then strain isolation substrates should be predictable based on which carbon sources can be utilized. We were able to predict 30% of strain isolations correctly, whereas using shuffled data as a null hypothesis, strain isolation source could only be predicted 15% of the time (Figure 6). In particular, the predictive value of the carbon utilization profile is well above our background estimates for isolations from dairy products, insects, plants, and water. This suggests that the environmental source of a strain shapes its carbon utilization profile.

## Discussion

We hypothesized that carbon utilization clusters in *Saccharomyces* may be the result of two possible mechanisms: (1) pleiotropy due to shared metabolic pathways or overlapping enzymes among carbon sources or (2) multi-trait coevolution due to similarities of carbon sources within environments. We did not find evidence that coordinated gain and loss of carbon source traits is the result of shared pathways or enzymes (clusters in Figure 3 vs. Figures 4A and 4B). In contrast, we found that a strain's set of carbon utilization traits often predicts the substrate from which the strain was originally isolated. This result suggests that a strain's environment determines its ability to use individual carbon sources. One important caveat, however, is that just because a strain was isolated from a particular habitat (e.g. beer or soil) does not mean that it typically grows on that source. Further, isolation of strains from similar sources may sometimes be confounded with shared phylogenetic history. In our data, strains isolated from similar substrates typically came from multiple species, therefore phylogenetic history is likely not a major confounder. This indicates that repeated parallel evolution of similar carbon utilization sets is due to common environmental pressures (e.g., [18]) across multiple strains and species of budding yeast. However, denser environmental sampling and phylogenetic analysis are required to better define the ecology of individual strains and genotypes.

Variation in the number and types of carbon sources available and used by a strain has the potential to affect both gene content and metabolic networks. This is because there are many genes that are likely to be affected by variation in carbon utilization phenotypes. For example, carbon sources are imported by diverse transport proteins [19], [20]. It has been demonstrated that there is an enrichment of duplicate genes in *S. cerevisiae* metabolism [4], [21]–[24], supporting the idea that gene copy number changes play an important role in the evolution of diverse metabolism. Ames *et al*. [10] analyzed variation in gene copy number among 39 strains of *S. cerevisiae* and 28 strains of *S. paradoxus* and found an enrichment of duplicates for genes with catalytic activity and sugar transport. Furthermore, they demonstrated that certain sets of over- and underrepresented duplicates correlate with adaptation to different environments.

Our results provide further support for how network structure can be impacted by the environment, suggesting that a wide metabolic breadth requires larger numbers of nodes, in the form of unique assemblages of specialized enzymes. Such networks will also be more expansive since most carbon sources are not funneled through a single pathway. These two factors suggest that metabolic networks change as a result of variation in metabolic breadth. The recent emphasis on molecular networks has received few rigorous tests about the impact of network structures on evolutionary processes [25], [26]. Our results indicate that metabolic network topology may not impose severe constraints on the evolution of carbon utilization phenotypes. Instead, our observation that traits are gained and lost independently of known metabolic network structure suggests that the networks themselves vary and evolve.
